# Understanding the Role of the Josephin Domain in the PolyUb Binding and Cleavage Properties of Ataxin-3

**DOI:** 10.1371/journal.pone.0012430

**Published:** 2010-08-26

**Authors:** Giuseppe Nicastro, Sokol V. Todi, Ezgi Karaca, Alexandre M. J. J. Bonvin, Henry L. Paulson, Annalisa Pastore

**Affiliations:** 1 National Institute for Medical Research, Medical Research Council, London, United Kingdom; 2 Department of Neurology, University of Michigan, Ann Arbor, Michigan, United States of America; 3 Science Faculty, Bijvoet Center for Biomolecular Research, Utrecht University, Utrecht, The Netherlands; University of Kent, United Kingdom

## Abstract

Ataxin-3, the disease protein in the neurodegenerative disorder Spinocerebellar Ataxia Type 3 or Machado Joseph disease, is a cysteine protease implicated in the ubiquitin proteasome pathway. It contains multiple ubiquitin binding sites through which it anchors polyubiquitin chains of different linkages that are then cleaved by the N-terminal catalytic (Josephin) domain. The properties of the ubiquitin interacting motifs (UIMs) in the C-terminus of ataxin-3 are well established. Very little is known, however, about how two recently identified ubiquitin-binding sites in the Josephin domain contribute to ubiquitin chain binding and cleavage. In the current study, we sought to define the specific contribution of the Josephin domain to the catalytic properties of ataxin-3 and assess how the topology and affinity of these binding sites modulate ataxin-3 activity. Using NMR we modeled the structure of diUb/Josephin complexes and showed that linkage preferences are imposed by the topology of the two binding sites. Enzymatic studies further helped us to determine a precise hierarchy between the sites. We establish that the structure of Josephin dictates specificity for K48-linked chains. Site 1, which is close to the active site, is indispensable for cleavage. Our studies open the way to understand better the cellular function of ataxin-3 and its link to pathology.

## Introduction

Parsimony in nature reveals itself in many ways. A particularly elegant biological example is the capacity to regulate divergent cellular pathways through the versatile properties of a single modifier protein. An excellent example of a multiple-function adaptor is ubiquitin (Ub), a small protein implicated in diverse cellular processes and pathways including transcription, protein degradation, cell cycle progression, viral infection, and immune response [1]. Ub represents a large molecular tag added posttranslationally to protein substrates, either as a single Ub molecule or as a polyUb chain in which Ub molecules are linked to one another through any of seven Ub lysine residues. PolyUb chains of different linkage type play distinct biological roles [2]. The best studied polyUb chain linkage, the K48-linkage, targets proteins for degradation by the proteasome [3]–[5], whereas K63-linked polyUb chains seem to serve a non-degradative role in transcription activation and DNA damage [6], [7]. Other chain linkages have also been identified, including branched and mixed-linkage chains, although their function has not been clarified [8]–[10].

Understanding how polyUb chains are recognized is an important, yet still poorly defined issue. It has been suggested that specificity is achieved by Ub chains interacting with multiple-domain proteins that contain different Ub binding motifs. One such protein is ataxin-3, a protein implicated in protein quality control pathways and transcriptional regulation [11]. Expansion of a polyglutamine (polyQ) tract in ataxin-3 causes the dominantly inherited neurodegenerative disease, Spinocerebellar Ataxia Type 3 (SCA3) or Machado-Joseph Disease (MJD). As a member of the MJD family of deubiquitinating enzymes (DUBs), ataxin-3 preferentially cleaves polyUb chains of four or more Ub subunits [12], [13]. K48-, K63-linked and mixed-linkage chains bind ataxin-3, but cleavage in vitro occurs preferentially for K63- and K48/K63-mixed linkage polyUb chains [14]. Ataxin-3 contains a catalytic N-terminal globular domain (the Josephin domain) with a cysteine protease fold, and a mostly unstructured C-terminal region that contains the polyQ tract and two or three Ub interacting motifs (UIMs), depending on the protein splice isoform [15]. In pull-down assays and surface plasmon resonance binding studies, the UIMs mediate Ub chain binding [14], [16]. We recently identified two additional Ub binding surfaces, both in the Josephin domain [17]. One, previously predicted on the basis of structural considerations, is close to the active site (site 1); the second Ub-binding site (site 2) coincides with the interaction surface of the Ub-like (Ubl) domain of hHR23B [18]. While the UIMs are clearly important in determining the cleavage specificity of the full-length protein, very little is understood about the role of the Josephin binding sites in chain specificity or cleavage. The potential importance of the two Josephin binding sites is suggested by their close proximity to the enzyme active site.

Here, we have investigated the structural constraints imposed by these two Ub-binding sites and their preferences in polyUb binding and in selecting the exact branching of Ub chains for cleavage. We compare the properties of Josephin and full-length ataxin-3 to cleave different polyUb chains (i.e. with K48, K63 or mixed linkages) and relate them to the binding and enzymatic properties of the domain. We observe a clear hierarchy between the two sites, which is dictated by their structural features and topological relationship. We also rationalize some of our results in terms of the structural constraints imposed by the Ub chains.

## Results

### Ub-binding sites 1 and 2 are not equally important for Ub chain cleavage by the isolated Josephin domain

**Figure 1:**
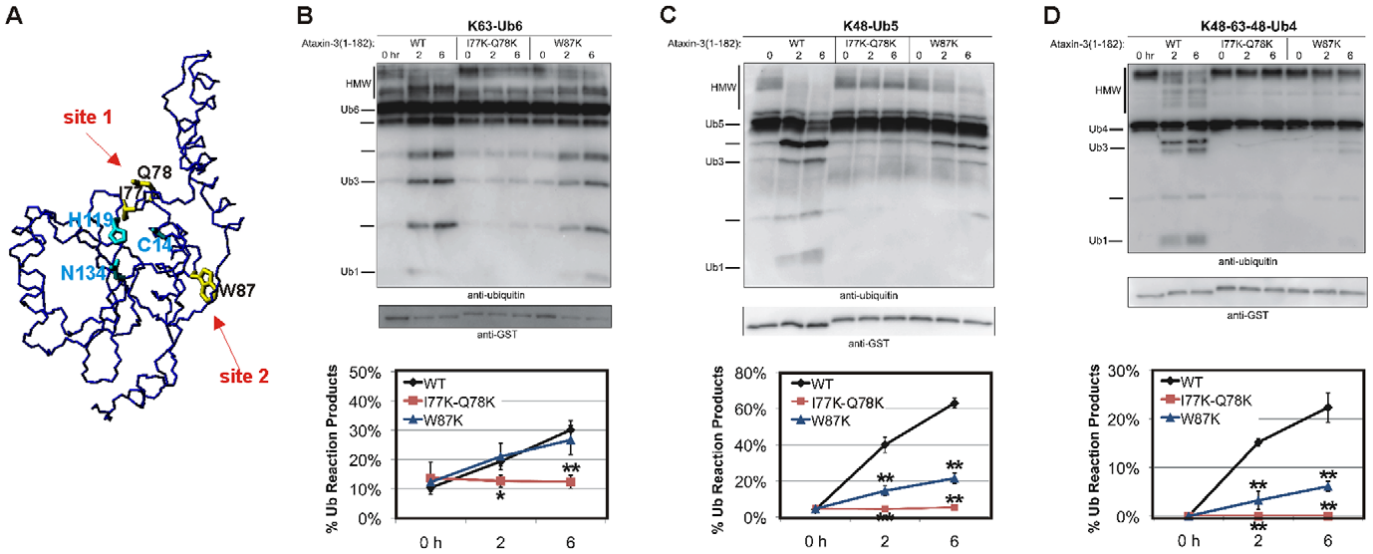
Relevance of Ub-binding sites 1 and 2 to polyUb chain cleavage by the Josephin domain. **A**) Josephin structure (1yzb). The side chains of the mutated residues and of the catalytic triad are indicated in yellow and cyan, respectively. Arrows denote the positions of the two Ub binding sites. **B–D**) GST-tagged, isolated Josephin domain samples of wild-type ataxin-3 (ataxin-3(1-182)-WT), mutated in Ub-binding site 1 (ataxin-3(1-182)-I77K-Q78K), or mutated in Ub-binding site 2 (ataxin-3(1-182)-W87K) were incubated with the following Ub chains: **B**) K63-linked hexa-Ub (K63-Ub6), **C**) K48-linked penta-Ub (K48-Ub5), or **D**) mixed-linkage tetra-Ub (K48-K63-K48-Ub4). Fractions were collected at the indicated times, electrophoresed on SDS-PAGE gels, and probed as indicated. HMW: high molecular weight Ub chain species that are thought to be dimers and trimers of the respective chains (14). Graphs below each blot represent semi-quantification of data from three independently run experiments. Shown are means +/− standard deviation. Asterisks: P<0.05 (*) or P<0.01 (**) when comparing reaction products from WT ataxin-3 to site-1 or site-2 mutated ataxin-3.

To examine the importance of Ub-binding sites 1 and 2 to the ability of ataxin-3 to cleave poly-Ub chains independently from the C-terminal UIMs, we carried out enzymatic Ub cleavage studies using the isolated Josephin domain (amino acids 1–182). We followed established protocols [14], [18]. In parallel we examined the cleavage properties of wild-type Josephin versus two mutants (Jos_I77KQ78K and Jos_W87K) that have been shown to individually abolish binding to site 1 or site 2, respectively [17] (**Fig. 1A**). Jos_I77KQ78K mutates two exposed residues that are close to the active site and are in the interface with the Ub molecule which binds in site 1. Jos_W87K mutates an exposed hydrophobic tryptophan found to be essential for binding both Ub and the Ubl of hHR23B to site 2 [18].

Our results show that Ub-binding site 1 is necessary for the Josephin domain to cleave K63-linked hexa and K48-linked pentaUb chains, as well as mixed-linkage chains (**Fig. 1B,C,D**). In contrast, site 2 shows a more nuanced involvement in Ub cleavage by the Josephin domain: site 2 is dispensable for cleavage of K63-linked chains (**Fig. 1B**), but mutating site 2 does lead to a reduction in cleavage of K48-linked and mixed-linkage polyUb chains (**Fig. 1C,D**).

These data indicate that site 1 is critically involved in any DUB activity of the Josephin domain, and suggest an involvement of site 2 in specific linkage recognition or preference by the Josephin domain.

### Ub-binding site 1 is also necessary for full-length ataxin-3 to cleave Ub chains

**Figure 2:**
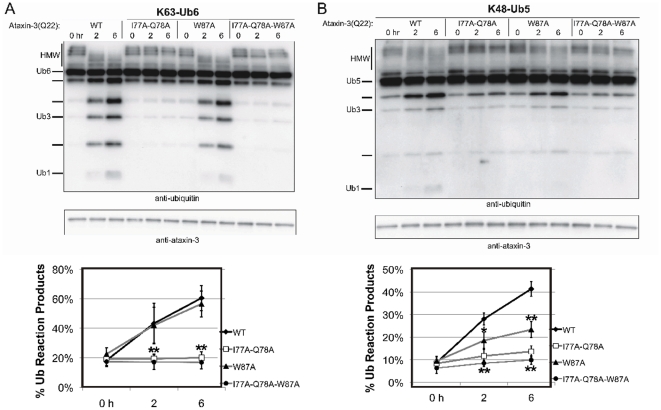
Relevance of Ub-binding sites 1 and 2 to polyUb chain cleavage by full-length ataxin-3. Full-length, untagged ataxin-3 protein samples that were wild type (ataxin-3(Q22)-WT), mutated in Ub-binding site 1 (ataxin-3(Q22)-I77A-Q78A), mutated in Ub-binding site 2 (ataxin-3(Q22)-W87A), or mutated in both sites (ataxin-3(Q22)-I77A-Q78A-W87A), were incubated with the following polyUb chains: **A**) K63-linked hexa-Ub (K63-Ub6), or **B**) K48-linked penta-Ub (K48-Ub5). Fractions were collected at the indicated times, electrophoresed on SDS-PAGE gels, and probed as indicated. Q22 denotes 22 glutamine residues in the polyQ region of ataxin-3. Lower panel for each blot represents semi-quantification of data from at least three independent experiments. Means +/− SD. Asterisks: P<0.05 (*) or P<0.01 (**) when comparing reaction products from WT ataxin-3 to ataxin-3 mutated at site 1, site 2 or both sites 1 and 2.

We next compared our results for the isolated Josephin domain with those obtained for full-length, non-expanded (Q22) ataxin-3. Independent of the Jos_I77KQ78K and Jos_W87K mutants tested above, we generated I77AQ78A and W87A mutants in full-length ataxin-3. Replacing the bulky hydrophobic residues in sites 1 and 2 with a smaller hydrophobic residue rather than a charged residue represents a milder mutation. Even so, we obtained results similar to those observed with the isolated Josephin domain: site 1 proved to be essential for the ability of full-length ataxin-3 to cleave K63-linked or K48-linked chains (**Fig. 2**). In contrast, site 2 mutations did not alter the ability of full-length ataxin-3 to cleave K63-linked chains but did impair cleavage of K48-linked chains.

Together, these results confirm the crucial importance of site 1 in cleaving all Ub chains and a role of site 2 in conferring linkage preference to ataxin-3.

### The isolated Josephin domain has clear preference for polyUb linkage and length

**Figure 3:**
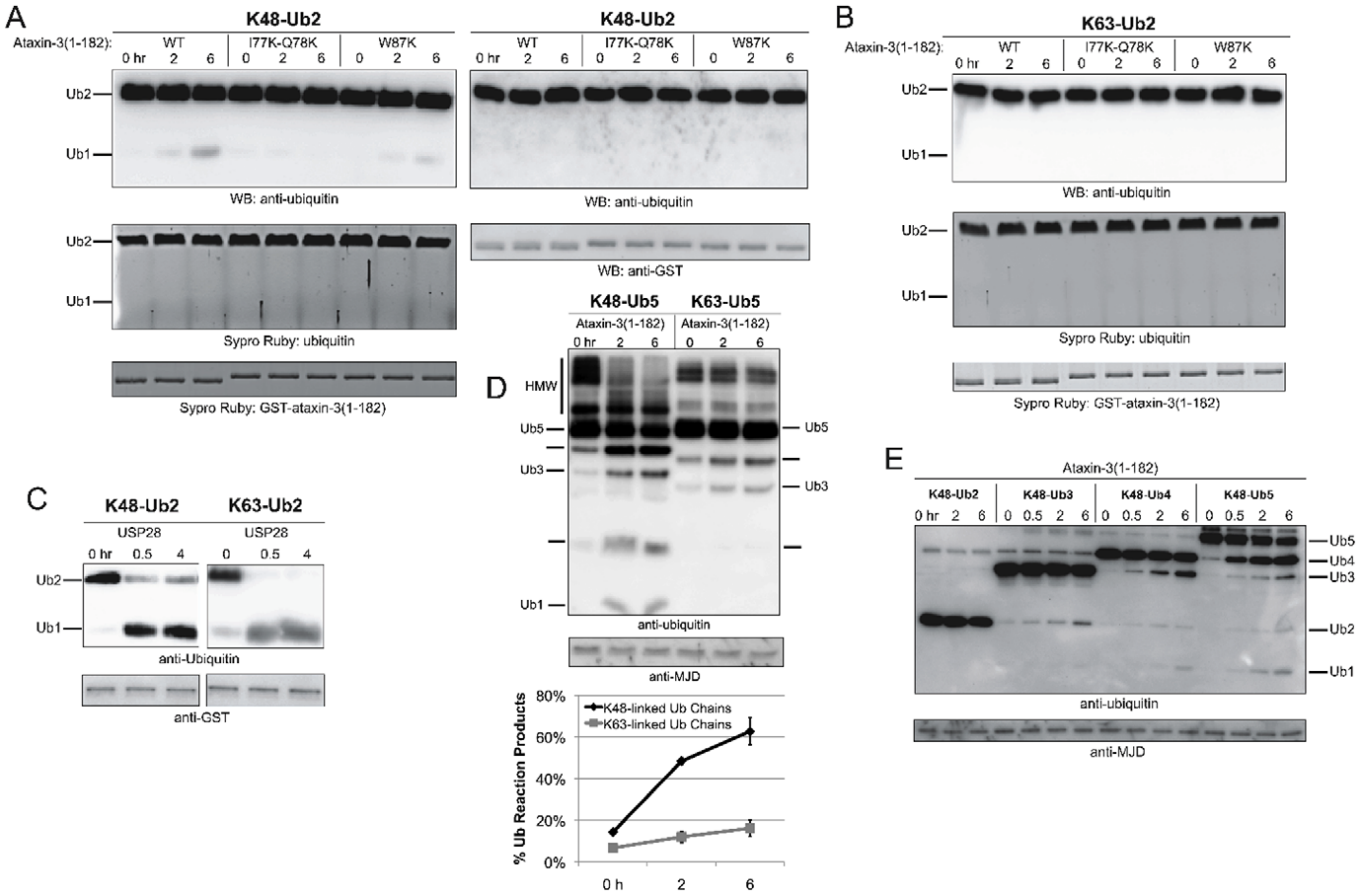
Cleavage of diUb chains by ataxin-3. **A**) Isolated Josephin domain species were incubated with K48-linked diUb chains (K48-Ub2) for the indicated times. Left and right panels are representative of independent trials with little or no detectable DUB activity, respectively. **B**) Isolated Josephin domain species were incubated with K63-linked diUb chains (K63-Ub2) for the indicated periods of time. **C**) GST-tagged USP28 was incubated with K48-linked or K63-linked diUb chains for the indicated times. **D**) Equal amounts of penta-Ub K48 or K63-linked chains (K48-Ub5; K63-Ub5) were incubated with the isolated Josephin domain. Fractions were collected at the indicated times. Lower panel: Quantification of data from the left panel and other similar experiments (N = 3). Shown are means +/− SD. **E**) Equal amounts of K48-linked di-, tri-, tetra-, or pentaUb chains were incubated with the isolated Josephin domain, and fractions were collected at the indicated time points.

To gather structural perspective into the role of sites 1 and 2 in Ub chain cleavage by the isolated Josephin domain, we first tested whether the Josephin enzymatic activity presents any preference towards polyUb chains of different lengths and/or linkage. We first incubated Josephin with either K48-linked or K63-linked diUb chains. We observed virtually no change in diUb levels or accumulation of monoUb in either case (Materials and Methods; **Fig. 3A, B**). The same behavior was observed for full-length ataxin-3 (data not shown). As a control, we verified that these diUb chains are cleavable by an unrelated DUB, USP28 [19]. In the presence of USP28, both K48- and K63-linked diUb chains were cleaved quickly and efficiently (**Fig. 3C**). These data indicate that diUb chains, whether K63- or K48-linked, are poor Josephin domain and ataxin-3 substrates.

To clarify linkage preferences of the Josephin domain in the absence of the C-terminal region of ataxin-3, we incubated isolated Josephin with equal amounts of K48-linked or K63-linked pentaUb chains (**Fig. 3D**). The isolated Josephin domain shows preference for K48 linkages compared to K63-linked chains, suggesting that the UIMs of ataxin-3 confer a protective effect toward K48-linked chains.

Finally, we systematically compared Josephin-mediated cleavage of K48-linked polyUb chains of increasing length. Longer chains are cleaved more efficiently than shorter ones, though in general the first cleavage at any length seems to be more efficient than successive ones (**Fig. 3E**).

### Isolated Josephin binds both K48- and K63-diUb chains but with different stoichiometry

**Figure 4:**
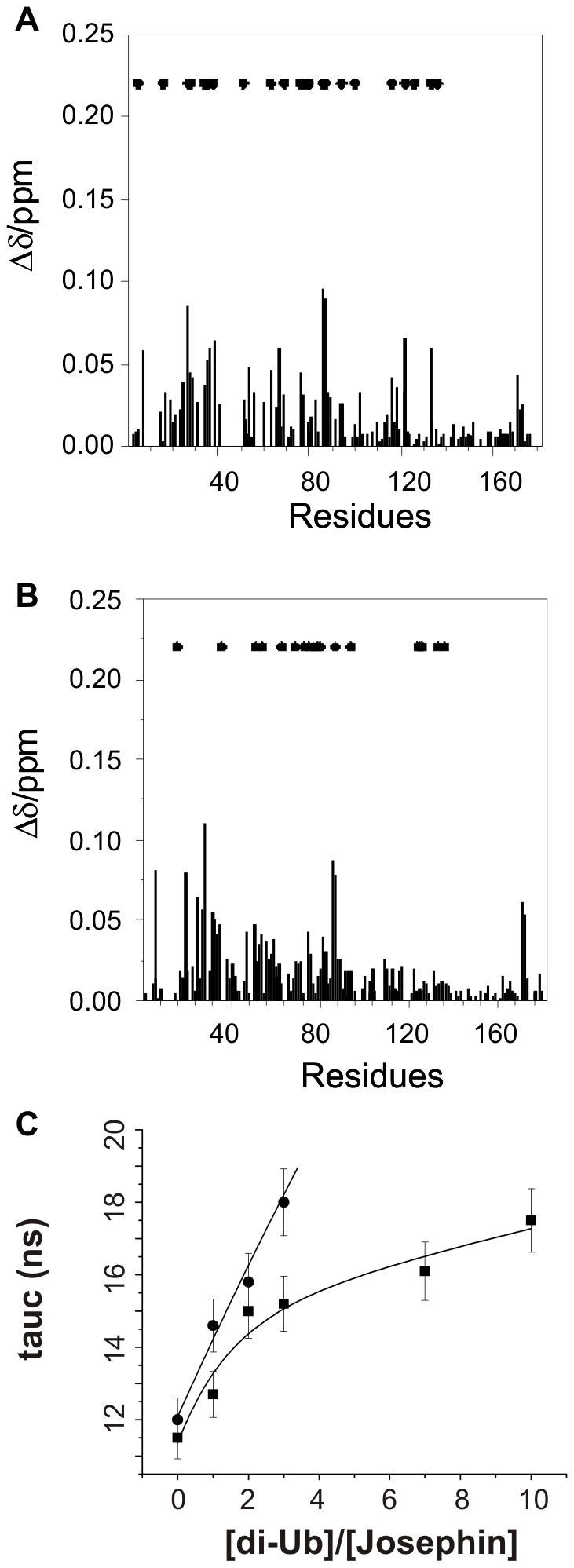
NMR titrations and models of Josephin/Ub complexes. **A**) and **B**) CSPs (Δδ) as a function of the amino acid sequences for the titration of the ^15^N labelled Josephin with unlabelled K48 (upper panel) and K63 (lower panel) diUb chains ([Josephin]/[diUb] 1/3). Asterisks denote residues whose resonances are attenuated upon binding. **C**) Comparison of the correlation times measured during titration of ^15^N labeled Josephin with K48- and K63-linked diUb chains.

To explain the above findings mechanistically, we sought to obtain a better structural description of the possible complexes involved. We used nuclear magnetic resonance (NMR) to study the effects of titrating the isolated Josephin domain with K48- or K63-linked diUb chains and to collect distance restraints which could be translated into structural models. A C14A mutant was used to be sure that no enzymatic cleavage could occur. We observed similar chemical shift perturbation (CSP) effects for both diUb linkages (**Fig. 4A,B**). Diagnostic CSP effects were those observed for the amide resonances of residues 27, 28, 86 and 87 which are in site 2, and of residues 16, 76, 77, 78, 122 and 133 which are in site 1 [18]. A small number of resonances disappeared or broadened significantly during the titration, indicating an intermediate exchange rate. Among these are 116 and 136 in site 1 (close to the catalytic triad) and 35, 36, and 38 in binding site 2.

We conclude that both Ub binding sites are populated during the titrations in agreement with what observed for monoUb [18]. Since, however, the two sites could be in principle occupied by Ub subunits from different polyUb chains, we clarified the stoichiometries by estimating the correlation times of the complexes by NMR. This is a very sensitive parameter that reflects directly the molecular weight of the species in solution. By reporting the correlation times as a function of different Ub:Josephin molar ratios, we observed that the complex with K48-linked diUb has always shorter values which tend to reach a plateau, albeit at high diUb:Josephin molar ratios (**Fig. 4C**). Close to plateau, the degree of saturation assuming dissociation constants around 30–60 µM [18] is ca. 85%. Under these conditions, the observed correlation time corresponds to a protein of 31–34 kDa, in excellent agreement with the molecular weight expected for a 1∶1 complex of Josephin with diUb (35 kDa). The complex with K63-linked diUb is instead always comparatively longer, and the curve does not seem to reach a plateau.

These results indicate that Josephin dictates preferential binding properties for different Ub linkages.

### Geometric features of Ub binding sites on Josephin determine binding specificity

**Figure 5:**
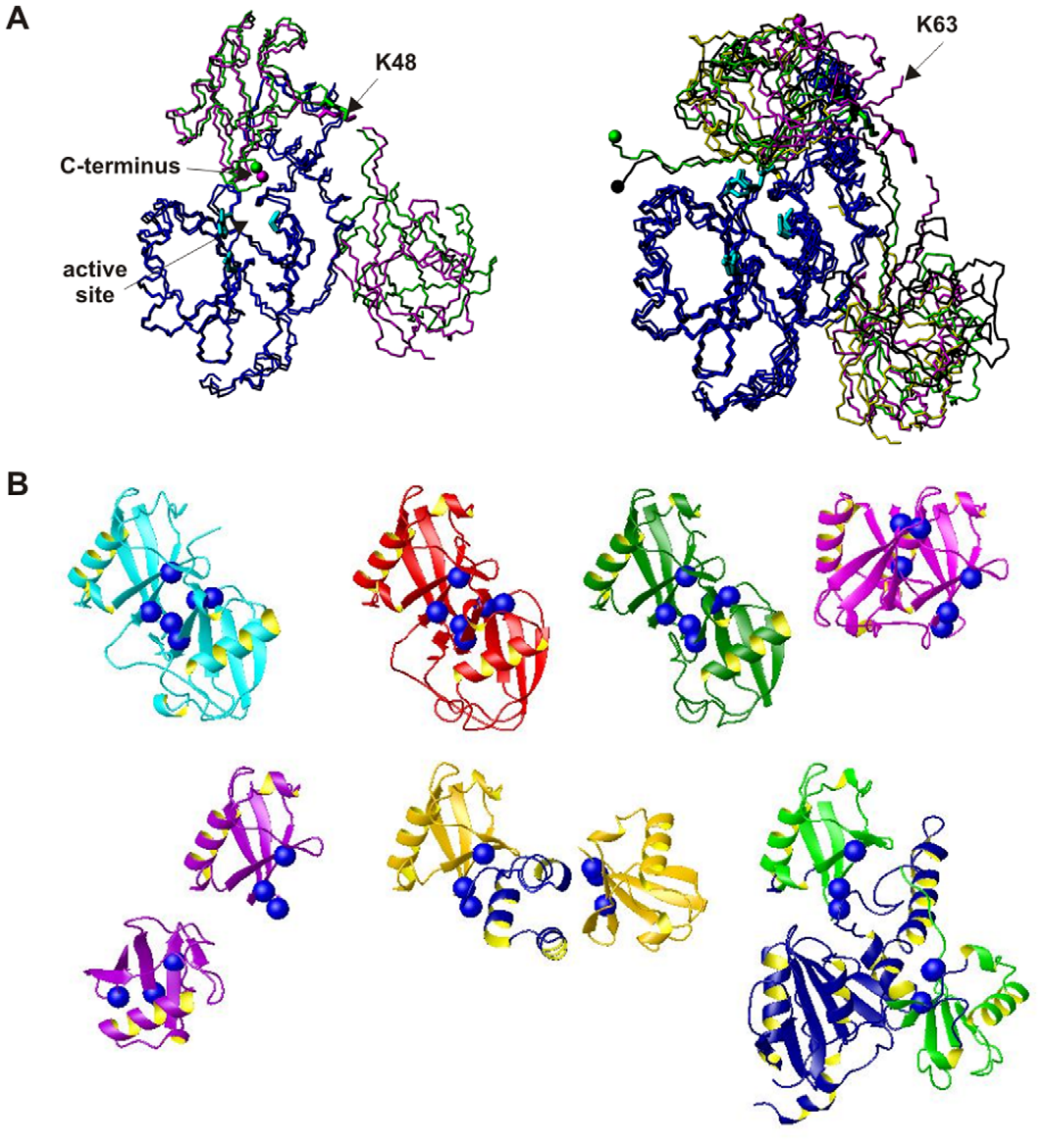
Modelling of diUb complexes with Josephin. **A**) Structural superposition of representative models of the Josephin complexes with K48- (left) and K63-linked (right) diUb. The structures are superposed on Josephin backbone atoms to enhance the similarities/differences of the Ub relative positions. The C-terminus (residue G76) of the Ub in site 1 is indicated by spheres. The side chains of the catalytic triad are shown in cyan. The side chains of the cross-linking lysines are also shown explicitly. **B**) Comparison of the Josephin/K48-linked diUb model and the known structures of polyUb chains in isolation and in a complex. Top line: the structures of diUb from Ub_2_ (1aar in cyan, 2bgf in red) and from Ub_4_ chains (2o6v in green, 1tbe in magenta). Bottom line: the structure diUb from a different crystal form (1f9j in purple); the structure of the UBA domain in complex with Ub_2_ (1zo6 in gold) and the structure of the Josephin complex (2jri, in blue). Residues L8, I44 and V71, which are often involved in Ub interfaces, are indicated by green spheres to provide a direct comparison of the relative orientations of the two subunits. With the sole exception of 1f9j, which was suggested to provide evidence of an open-to-closed equilibrium, the Ub subunits are in a closed conformation when not in a complex. In the two complexes, the conformation is open with the linkers differently stretched to adapt to the binding sites.

To rationalize these observations, we translated the distance information into molecular models using the biomolecular docking program, HADDOCK [20], [21]. We performed three docking runs based on the NMR CSP data. In the first run, we imposed a K48-diUb linkage in combination with the ambiguous interaction restraints (AIRs) defined from the CSP data. This resulted in two ensembles of solutions with similar scores (**Fig. 5A**). Site 1 (or proximal) Ub shares the same orientation in all solutions, suggesting that this site is overall better defined. Upon binding, the hairpin bends on one side to allow space for the Ub linkage and wraps around the surface of proximal Ub (i.e. the Ub in site 1 which has the C-terminus free) which contains the β-sheet. The bending is much more pronounced than in free Josephin [18], supporting the hypothesis that this secondary structure element helps determine binding specificity. Two contiguous binding surfaces seem instead to be equally compatible with the experimental restraints for Ub binding to site 2 (distal). One surface is similar to that observed in the Josephin/mono-Ub complex [17]; the other is formed by residues in the β1/β2 and α/β3 loops. In both clusters, the aromatic Josephin side-chains of Y27, F28 and W87 contribute to the interface. Strikingly, both clusters contain solutions with the C-terminus of site 1 Ub at close proximity to the Josephin active site, even though no explicit distance restraints were defined to position a Ub close to the active site of Josephin. Comparison of the model of the K48-linked diUb/Josephin complex with other diUb structures shows that, to bind both sites, the diUb chain needs to have an extended linker, adopting a conformation much more open than that observed for the K48-linked diUb complex with the UBA domain of HHR23A [22]. K48-linked polyUb chains are known to exist in solution as a fast dynamic equilibrium between open and closed conformations [23], [24] (Fig. 5B). The closed conformation is predominant at neutral pH and in the absence of binding partners. Other diUb complexes do not, however, show the open conformation necessary to accommodate Josephin.

In the second run, the same docking procedure was followed but this time imposing a K63 Ub linkage (**Fig. 5C**). The solutions were more scattered and no biologically meaningful structures could be obtained (i.e. none having the Ub C-terminus close to the catalytic center). Even in the best case (cluster 2), the C-terminus of site 1 Ub is close only to H119 but not to C14 or N134. To investigate if this could be an artifact of our docking procedure we repeated the calculations with an additional distance restraint to position the C-terminus of the site 1 Ub into the Josephin active site. No solution was found to satisfy this restraint (data not shown) indicating that the two subunits of a K63-linked diUb cannot be accommodated simultaneously in both Josephin sites.

Finally, we performed a run in which the linkage preference was left ambiguous by defining a linkage restraint including both K48 and K63. Although in principle both linkage types were obtained at the rigid-body docking stage, the only selected solutions for the subsequent semi-flexible refinement correspond to the K48 linkage.

These results indicate that the relative position of the Ub binding sites on Josephin dictate different binding specificities. The structure of K63-linked diUb is incompatible with the geometric requirements imposed by the relative position of the two Josephin binding sites and that only K48-linkage diUb is able to occupy both Ub-binding sites of Josephin simultaneously.

### hHR23A influences the ability of Josephin domain to cleave polyUb chains

We tested whether the presence of hHR23A has any effect on ataxin-3 cleavage of polyUb chains. Ataxin-3 is known to interact in cells and in vitro with the Ubl domain of hHR23A [18], [25], [26]. This interaction is centered on residue W87 of ataxin-3, which is also the critical residue for Ub binding by site 2 [17]. Consistent with this, HHR23B Ubl was shown to compete monoUb in vitro [18].

**Figure 6:**
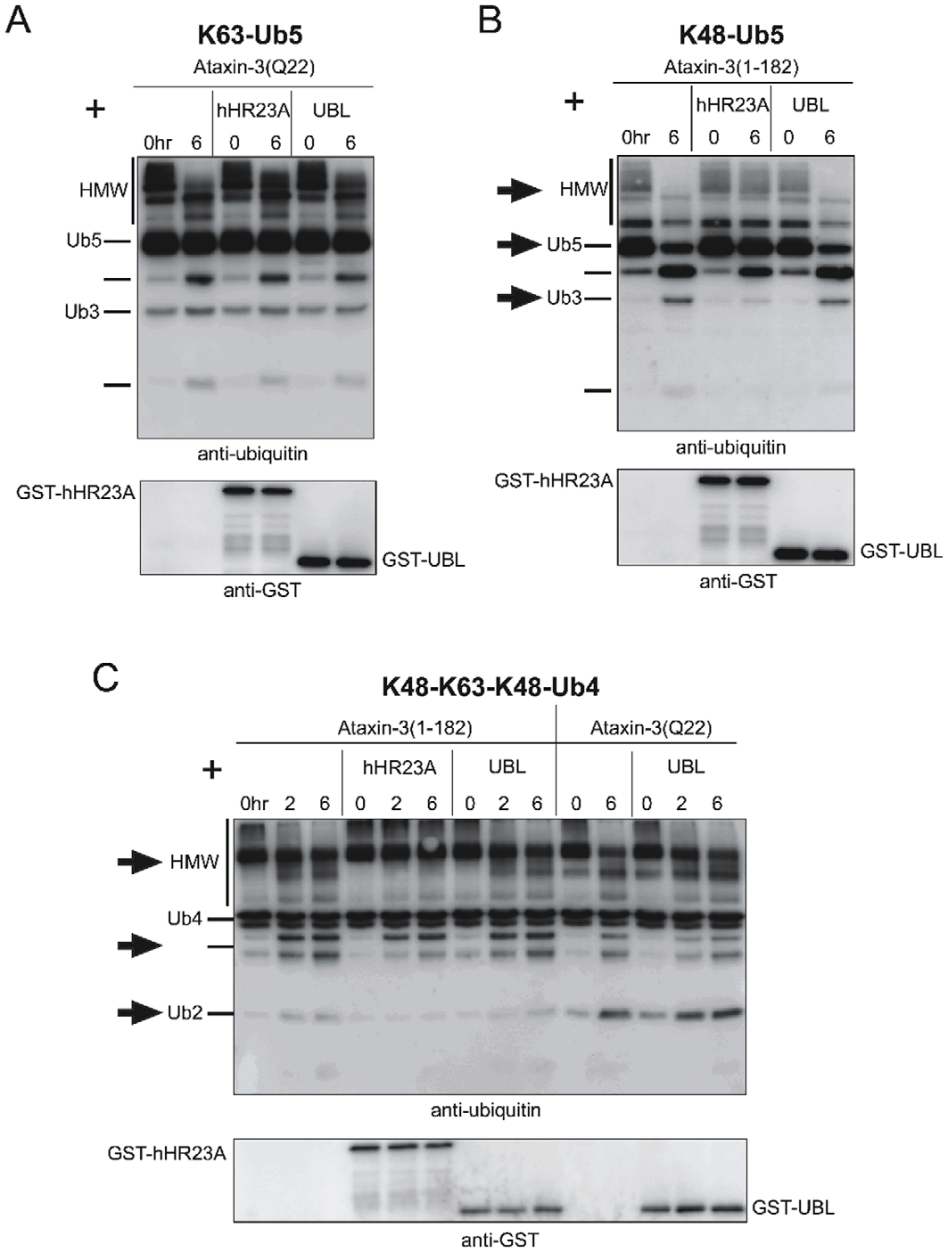
hHR23A influences cleavage of polyUb chains by the Josephin domain. **A)** Full length ataxin-3 (ataxin-3(Q22); 100 nM) was incubated with K63-Ub5 (250 nM) chains and either full length GST-tagged hHR23A (100–250 nM) or its GST-tagged UBL domain (100–250 nM). Fractions were collected at the indicated times. **B**) Isolated Josephin domain (ataxin-3(1-182); 100 nM) was incubated with K48-Ub5 chains (250 nM) and either GST-tagged hHR23A (100–250 nM) or its GST-tagged UBL domain (100–250 nM). Samples were collected at the indicated times. Block arrows highlight differences in cleavage activity among samples. **C**) Full length ataxin-3 (ataxin-3Q22(WT); 100 nM) or the isolated Josephin domain (ataxin-3(1-182); 100 nM) were incubated with mixed-linkage polyUb chains (K48-K63-K18-Ub4; 250 nM) and either full-length GST-hHR23A (100–250 nM) or its GST-tagged UBL domain (100–250 nM). Block arrows highlight differences in cleavage among samples.

The ability of ataxin-3 to cleave polyUb chains is not visibly affected by the presence of either full-length hHR23A or its isolated Ubl domain (**Figs. 6A and C**). However, full-length hHR23A does reduce the ability of the isolated Josephin domain to cleave polyUb chains (**Figs. 6B and C** **,** block arrows). In contrast, the isolated Ubl domain of hHR23A does not affect cleavage by the isolated Josephin domain.

Together, these data argue against a significant impact of hHR23A/ataxin-3 interaction on the ability of full length ataxin-3 to cleave chains in this specific setup. This becomes particularly evident when considering that the Ubl domain of hHR23A, which interacts with ataxin-3, does not have a detectable effect on Ub cleavage.

## Discussion

We previously showed that full-length ataxin-3 binds both K48- and K63-linked Ub chains, yet preferentially cleaves K63 linkages in a manner dependent on the UIMs present in the C-terminus of ataxin-3 [14]. More recently we showed that the amino-terminal catalytic Josephin domain also contains two distinct Ub binding sites in addition to the 2-3 UIMs contained in the C-terminus [17]. Central questions directly following from these previous findings are therefore: What is the specific role of Josephin in cleavage? And what is the relationship and mutual influence of the multiple Ub binding sites?

Here, we addressed these questions by dissecting the relative importance of the Ub binding sites by comparing the behaviour of the isolated Josephin with that of the full-length protein. In addition to being important for the non-pathologic functions of the protein, Josephin has been shown to determine the aggregation properties of non-expanded ataxin-3 in vitro [27]–[29], although still little is known about its role in pathogenesis. Isolated Josephin species could be generated by alternative splicing or proteolysis. Cleavage products of expanded ataxin-3 have been reported in cell, fruit fly and mouse models of SCA3 and in SCA3 disease brains [30]–[33], but most of the described fragments are C-terminal portions of ataxin-3 containing the polyQ domain. N-terminal cleavage products that include the catalytic triad would have the capacity to act as a functional DUB whose properties and chain selectivity would differ in some respects from that of full length ataxin-3. Thus, N-terminal Josephin domain fragments potentially could influence SCA3 pathogenesis by affecting the balance in Ub species in patients [34]. Whether this indeed contributes to SCA3 disease pathogenesis is currently unknown and certainly deserves future attention.

Independently from a role in pathogenesis, a comparison between full-length ataxin-3 and isolated Josephin can help us to understand the relative influence of different and distal regions of the protein both on its normal function and on disease pathogenesis. Through a combined use of rationally designed mutations of the Josephin domain and a detailed comparison of the enzymatic properties of the domain with those of full-length ataxin-3, we confirm that, in the absence of other competitors, Ub chains are able to bind both Ub binding sites of Josephin. The structure of Josephin does, however, dictate the stoichiometry of binding and therefore preferentiality for diUb linkages: the two subunits of K48-linked diUb can populate both sites simultaneously whereas K63-linked diUb chains cannot.

The importance for enzymatic activity of the two binding sites differs greatly. Site 1, which is contiguous to the active site, is necessary for cleavage as mutations of critical residues in this site to either lysine or alanine abolish cleavage both by the isolated Josephin domain and by full-length ataxin-3. Its role must therefore be that of anchoring the Ub molecule and allowing correct positioning of the Ub C-terminus into the active site as observed in our models. In contrast, when site 2 is mutated we observe only modest differences in the cleaving pattern of Josephin. Site 2 thus seems to be comparably less important and disposable for cleavage.

These observations suggest two different hypotheses to explain the role of site 2. According to the first one, site 2 would modulate the properties of site 1 but would be significantly populated only in full-length ataxin-3, where correct positioning of polyUb chains would be determined by the UIMs. It has been shown experimentally that the two isolated, contiguous ataxin-3 UIMs, which are connected by a two residue linker, contribute to binding of diUb with binding constants around 100 µM and no obvious linkage preferences [16]. Their role could become dominant in full-length ataxin-3, where they are likely to provide cooperativity and anchor the polyUb chains. The unstructured C-terminus, in which all three UIMs reside, would easily act as a flexible tethering arm which brings the anchored Ub chains to the catalytic site to be positioned correctly. The UIMs could also play a determinant role in chain specificity since full-length ataxin-3 preferentially cleaves K63-linked polyUb [14] whereas we have demonstrated here that the Josephin domain alone preferentially cleaves K48-linked polyUb (**Fig. 3**). This model is further supported by the evidence that mutating the UIMs enhances the ability of ataxin-3 to cleave K48-linked polyUb chains [14].

A different and more drastic hypothesis is that site 2 is never populated by Ub but rather by the Ubl of hHR23A and hHR23B or Ub-like domains in other proteins. Consistent with this, we do not see significant effects on cleavage when site 2 is saturated with hHR23A Ubl. This is also in agreement with the tighter affinity of Josephin with the highly homologous Ubl of hHR23B (12 µM) as compared with the K_d_ of ∼50 µM for the complex with monoUb [17]. The presence of other domains of the hHR23 proteins would also contribute to increase the binding affinity of this protein for ataxin-3. This possibility has important consequences regarding our understanding the functions of ataxin-3, particularly in a cellular context. A complete understanding of ataxin-3′s interactions with polyUb awaits, at this point, a structural characterization of full-length ataxin-3 in complex with Ub chains. The unstructured C-terminal portion of ataxin-3 currently presents a formidable challenge for such studies.

Finally, we observed that, as for full-length ataxin-3 [14], the isolated Josephin domain prefers to cleave longer polyUb chains. The pattern of products generated by cleavage of polyUb chains indicates that terminal Ub-Ub linkages are cleaved more easily than internal ones. It is uncertain how this occurs at the molecular level, but this observation suggests that the Josephin domain may distinguish terminal vs. internal Ub-Ub linkages either as a single molecule or by interacting with other Josephin domains.

In summary, we have shown that the catalytic domain of the deubiquitinating enzyme and polyQ disease protein, ataxin-3, has a preference for interacting with K48-linked diUb chains compared to K63-linked diUb chains. Ub-binding site 1 on the Josephin domain is indispensible for the enzymatic activity of ataxin-3, while site 2 may be dedicated to the recognition of hHR23 proteins. Our data provide significant new insights into ataxin-3′s interactions with its substrates and the manner in which isopeptide bonds are positioned for cleavage.

## Experimental procedures

### Site-directed mutagenesis

The following primers were used to generate missense mutations of specific residues of ataxin-3 into alanine (Quickchange Mutagenesis, Stratagene): 5′gccttctggaaatatggatgacagtggttttttctctgctgcggttataagcaatgccttg (forward) and 5′caaggcattgcttataaccgcagcagagaaaaaaccactgtcatccatatttccagaaggc (reverse) for the Iso77-Ala/Glu78-Ala mutant, 5′ggttataagcaatgccttgaaagttgcgggtttagaactaatcctg (forward) and 5′caggattagttctaaacccgcaactttcaaggcattgcttataacc (reverse) for the W98A ataxin-3 mutant. Mutations of the equivalent Josephin residues into lysines were obtained as previously described [17]–[18], [35]. We observed similar results in our enzymatic assays whether these specific residues were mutated to lysine or to alanine. The primers: 5′actatccatggagtccatcttccacgagaaacaagaaggctcacttgctgctcaacattg (forward) and 5′atcttgcggccgcttacctaatcatctgcaggagttggtcagcttcgcaatctggcagatcacc (reverse) were used for a C14A Josephin mutant (cloned using NcoI and NotI restriction sites).

### Protein production

The proteins were expressed in E. coli BL21(DE3) and purified as previously described [35]. Labeled proteins were obtained by growing E. coli in synthetic medium containing ^15^NH_4_Cl as the sole source of nitrogen. The K48-linked diUb used for structural studies was produced as described elsewhere [36]. Briefly, recombinant Ub mutants (UbD77 and UbK48C) were produced and incubated overnight with E1 (Boston Biochem), Cdc34, ATP, phosphocreatine, creatine phosphokinase and inorganic pyrophosphate at 37°C. The protein was then purified using a monoS cation exchange column after stopping the reaction by adding acetic acid at pH 4.5. K63-linked diUb chains were generated with the same protocol but using UbD77 and UbK63R mutants and replacing E2 with Ubc13 and Uev1a enzymes. The purity and chemical identity of the resulting proteins were examined by SDS polyacrylamide gel electrophoresis and mass spectrometry.

### Enzymatic assays

PolyUb chains (250–1000 nM; Boston Biochem) were incubated with full-length ataxin-3 or isolated Josephin domain species (100–400 nM) in DUB buffer (50 mM HEPES, 0.5 mM EDTA, 1 mM DTT, 0.1 mg/ml ovalbumin, pH 7.5) at 37°C. Fractions were collected in 2% SDS, 100 mM DTT sample buffer, boiled for 1 min and electrophoresed in 15% SDS-PAGE gels or in 4%–20% Ready Gels (BioRad). SDS-PAGE electrophoresis and western blotting were conducted as previously described [14], [37]. In-gel protein staining by Sypro Ruby (Invitrogen) was conducted per the manufacturer's instructions, and imaged in a VersaDoc MP5000 imager (BioRad) with UV trans-illumination. Rabbit anti-Ub (1∶500; DAKO), mouse anti-ataxin-3 (1H9, 1∶1000; generous gift from Yvon Trottier), rabbit anti-MJD (1∶20,000) [38], goat anti-GST (1∶20,000) (GE Healthcare) antibodies were used for protein detection. High molecular weight (HMW) ubiquitin species observed by western blotting in enzymatic assays are believed to be multimers of the respective substrates [14].

Although we routinely observe monoUb generated during our enzymatic assays, monoUb is less readily detected by western blotting techniques than longer Ub chains. Thus, for diUb cleavage assays, to ascertain that diUb is indeed not a substrate for ataxin-3 we used a combination of western blotting and in-gel protein detection (as described above), as well as positive controls with the unrelated DUB USP28 with which the monoUb reaction product is easily detected. This approach, together with data from cleavage of polyUb chains by ataxin-3 (in which we detect monoUb) ensures us that the absence of monoUb from diUb cleavage assays is due to the actual absence of monoUb generated during the reaction rather than to a lack of detection on immunoblots.

### Western blotting quantification and statistical analyses

For semi-quantification of western blots, images were collected below-saturation levels using VersaDoc 5000MP (BioRad). Quantification was conducted with Quantity One (BioRad). Background was subtracted equally among all lanes and the intensity of each band was integrated as the area under each intensity curve. The sum of intensities of all product bands was divided by the sum of intensities of all bands in that lane. For example, for cleavage of Ub5 chains, the equation was: Σ(I_Ub4_, I_Ub3_, I_Ub2_, I_Ub1_)/Σ(I_Ub5_, I_Ub4_, I_Ub3_, I_Ub2_, I_Ub1_). Statistical analyses of data collected from independent experiments were conducted using two-tailed student t-tests. All graphical data are shown as means +/− standard deviation.

### Structure calculations

For structure calculation purposes the biomolecular docking program, HADDOCK [20], [21] was used based on CSP data analyzsed with a recently developed method, SAMPLEX [39]. SAMPLEX identifies the residues that reflect significant chemical shift changes through an unbiased and automated methodology; it outputs three types of residues: residues classified as unaffected (core family), interfacial (interface family) and intermediate. SAMPLEX was run to determine the significant changes in the CSP data of the two binding sites obtained from NMR titrations of Josephin constructs with a mutation at either site 1 (I77K-Q78K) or site 2 (W87K). The active residues for docking were defined based on the SAMPLEX selection (including the intermediate class for Josephin to increase the possible interface region). For Josephin, the solvent accessible neighbors of the active residues within a 10 Å cut-off were selected as passive residues in order to increase the ambiguity, resulting thus in a larger interface coverage. Passive residues on the Ub surface were defined in the same manner using a 5 Å cut-off.

Three different three-body (two Ub molecules and Josephin) docking runs were performed to test the ability of K48 and K63 linked diUb to interact with Josephin: K48 or K63 linkages were imposed in two of them. In a third run, ambiguous linkage restraints were imposed to both K48 and K63 to see which one would be preferentially selected during docking. In all three runs, 5000 rigid-body docking solutions were generated and the best 200 were subjected to semi-flexible refinement in torsion angle space followed by refinement in explicit water. Random removal of restraints was turned off (noecv = false) and additional center-of-mass restraints were defined between the various molecules to ensure compact solutions. All other parameters were left to their HADDOCK default values. The C-terminal tail of each Ub was defined as fully flexible together with K48 and/or K63 depending on the linkage type.

The isopeptide bond between the two Ubs was defined by imposing a loose distance restraint (10 Å) between the side chain ammonium group of either K48 or K63 and the carboxyl atom of G76. In a third docking run with an ambiguous linkage definition, an ambiguous distance restraint was defined including both K48 and K63. A final additional water refinement was performed in which the distance restraint was decreased to 1.5 Å in order to re-establish the iso-peptide bond.

To check if positioning of Ub into the catalytic site could be consistent with the measured chemical shift data, the three runs were repeated with one additional distance restraint (10 Å) between the Josephin side chain sulphur of C14 and the C-terminal carbon of site 2. This distance was shortened to 5 Å for the additional water refinement step.

The resulting models of each docking run were clustered with a 5 Å cut-off and the clusters ranked based on the average HADDOCK score calculated over the best four members of each cluster.

### Estimate of the complex stoichiometries

NMR experiments were performed at 298 K on a Bruker Avance spectrometer operating at 600 MHz and equipped with cryoprobe. Titrations of ^15^N labelled Josephin or C14A Josephin mutant (0.05 mM in 20 mM sodium phosphate pH 6.5, 2 mM DTT) with diUB chains were performed with stepwise additions of concentrated stock solutions of K48-linked or K63-linked diUb chains (1.47 mM and 0.4 mM, respectively) up to a 10 and 2.5 fold molar excess, respectively. Combined measurements of T1 and T2 relaxation times were performed to estimate the correlation times using a home modified Bruker sequence (hsqct2etf3gpsi3d). Overall correlation time values were estimated from the R2/R1 ratio using the program R2R1_TM available from the website of A.G. Palmer's group (A.G. Palmer III, Columbia University) [40]. The values were compared to a literature calibration curve [41].
